# Rhinocerebral Mucormycosis with Extensive Cranial Nerve Palsies in a Diabetic Patient

**DOI:** 10.7759/cureus.50451

**Published:** 2023-12-13

**Authors:** K.V.P. Munasinghe, F.H.D.S. Silva

**Affiliations:** 1 General Medicine, Colombo South Teaching Hospital Kalubowila, Colombo, LKA; 2 Medicine, Faculty of Medical Sciences, Colombo South Teaching Hospital Kalubowila, University of Sri Jayewardenepura, Colombo, LKA

**Keywords:** immunocompromised, sinusitis, amphotericin, diabetes, cranial nerve palsy, rhinocerebral mucormycosis

## Abstract

Mucormycosis is a common opportunistic fungal infection with a disseminated nature. Despite being a devastating disease with the involvement of multiple upper cranial nerves, the implications of the seventh cranial nerve have been infrequently encountered. Although the radiological evidence with sinus destruction supports the diagnosis, histological specimen showing fungal elements confirms it as fungal culture doesn’t always demonstrate a high diagnostic yield. Early detection and multimodal treatment are mandatory to prevent detrimental outcomes and to control the disease progression. We, hereby present a rare case of rhinocerebral mucormycosis with multiple cranial nerve involvement including the facial nerve in a 70-year-old female with long-standing poorly controlled diabetes mellitus.

## Introduction

Mucormycosis is a fungal infection caused by a group of saprophytic fungi (molds) frequently found in soil, decaying matter, and the upper respiratory tract of healthy people [1]. Mucormycosis is an aggressive disease with a disseminated nature out of which rhinocerebral disease with cranial nerve involvement signifies its severity. The majority (96%) of the patients with mucormycosis carry an underlying risk factor and diabetes mellitus, particularly with ketoacidosis has become the leading cause (70%) out of all [2].

A few other risk factors include the usage of immunosuppressive drugs, immunosuppressive states like malignancies and post-solid organ and hematopoietic stem cell transplantation, injection drug use, infections like coronavirus disease (COVID-19), acquired immunodeficiency syndrome (AIDS), and soft tissue trauma. A controversy has been demonstrated in iron overload status being a risk factor for the disease pathogenesis and treatment with certain iron chelators also contributing to that [3]. We hereby present a case of rhinocerebral mucormycosis with extensive cranial nerve palsies in a diabetic patient.

## Case presentation

A 70-year-old woman with diabetes mellitus type two for 15 years presented with a two-day history of drooping of the left eyelid with epistaxis and mouth deviation to the right side. There was no history of fever. She did not give a history of COVID-19 infection in the past nor was there any ongoing infection as evidenced by the negative COVID-19 polymerase chain reaction (PCR) test. She was vaccinated with two doses of COVID-19 (Sinopharm) by the time of the presentation to the hospital. Throughout the last 15 years, her glycemic level had been poorly controlled evidenced by serial glycemic level measurements (fasting blood sugar and HbA1C) far beyond the reference values and other microvascular complications such as diabetic retinopathy, neuropathy, and nephropathy for which necessary interventions had not been considered. She was on oral hypoglycemic drugs with maximum doses namely metformin 1 g twice daily, gliclazide 160 mg twice daily, and sitagliptin 100 mg mane with poor compliance.

On examination, the patient was afebrile and mildly dehydrated. The pulse rate was 102/min, the blood pressure was 130/85 mmHg. Cranial nerve examination revealed involvement of 1st to 7th cranial nerves on the left side. There was reduced olfactory sensation bilaterally. Involvement of the left optic nerve was evidenced by complete blindness of the left eye with chemosis without proptosis with a 4 mm pupil with sluggish light reflex in the absence of papilledema. The right eye was normal with a 2 mm reactive pupil. A complex ophthalmoplegia could be appreciated. Along with that, weakness of the oculomotor, trochlear, and abducens nerves of the left side was present. Left-sided facial sensory loss along the distribution of the ophthalmic and maxillary divisions of the trigeminal nerve was detected. She also had a left-side lower motor neuron type facial nerve palsy. Her limb examination and other systems examination including cardiovascular, respiratory, and abdominal systems were unremarkable. The middle turbinate of the nasal cavity was necrotic with impacted blood clots. The right-side nasal cavity was devoid of debris at the time of examination.

**Figure 1:**
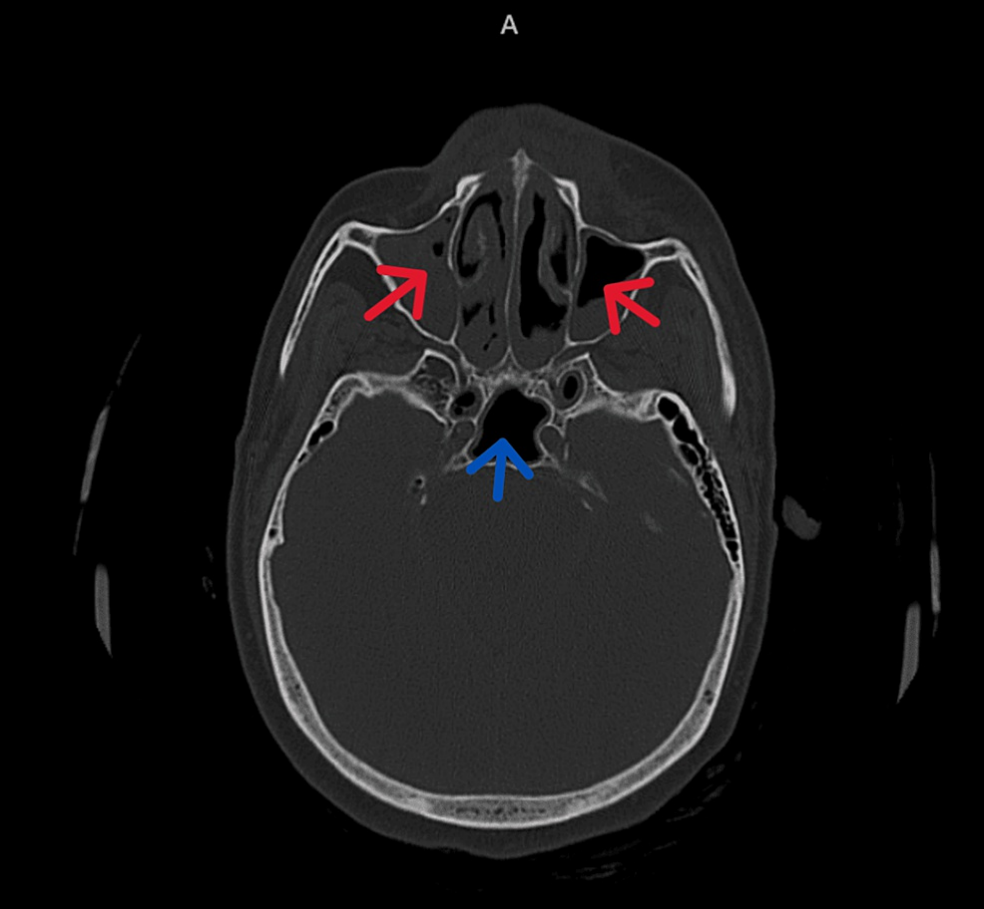
Non-contrast CT (NCCT) brain (bone window) showing maxillary (red arrow) and sphenoidal sinuses (blue arrow) Near complete opacification of right maxillary sinus with mild sclerosis of the bony walls. Mucosal thickening of left maxillary sinus and bilateral sphenoid air cells.

**Figure 2:**
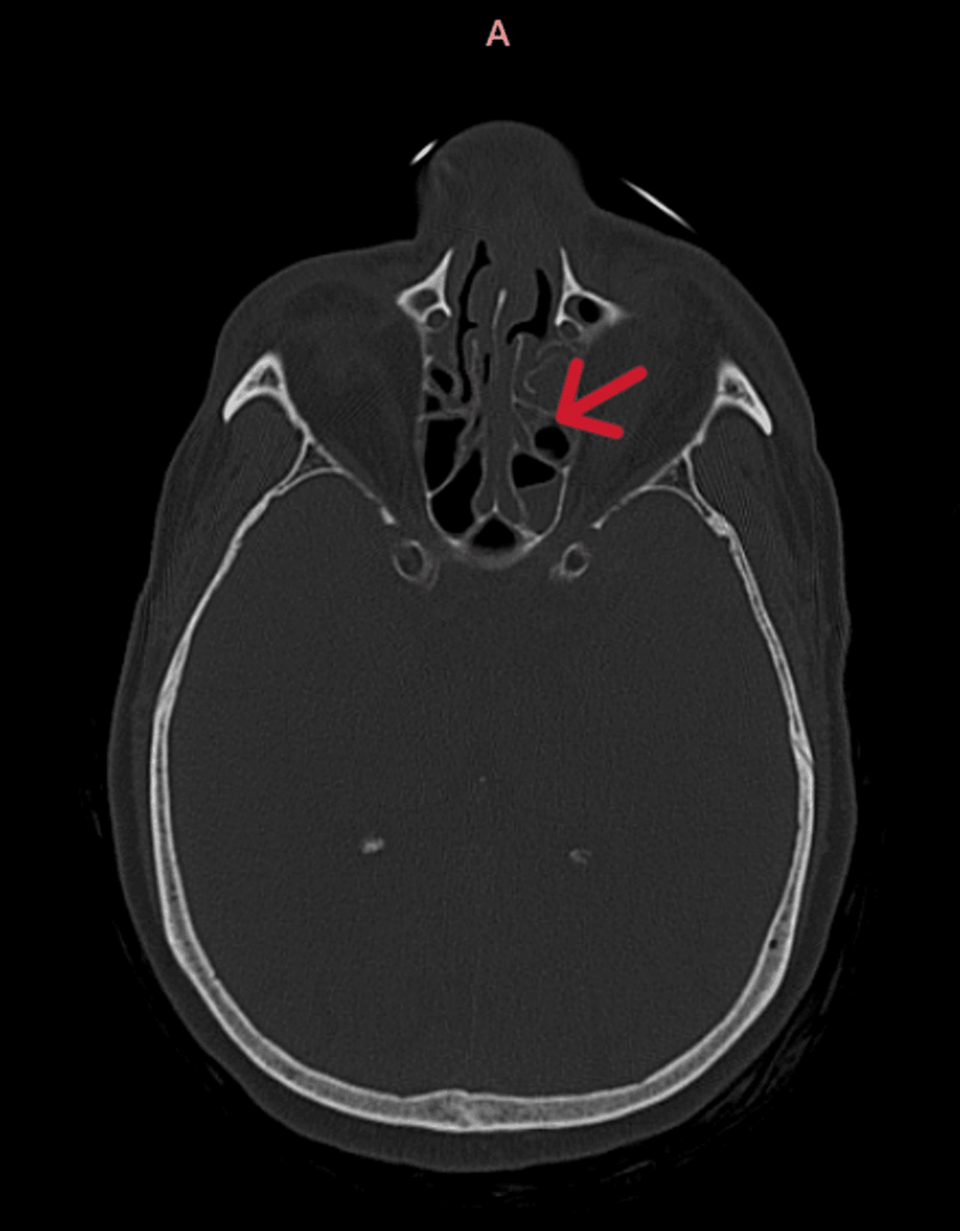
Non-contrast CT (NCCT) brain bone window showing ethmoidal air cells (red arrow) Mucosal thickening of bilateral ethmoidal air cells is seen in this scan.

**Figure 3:**
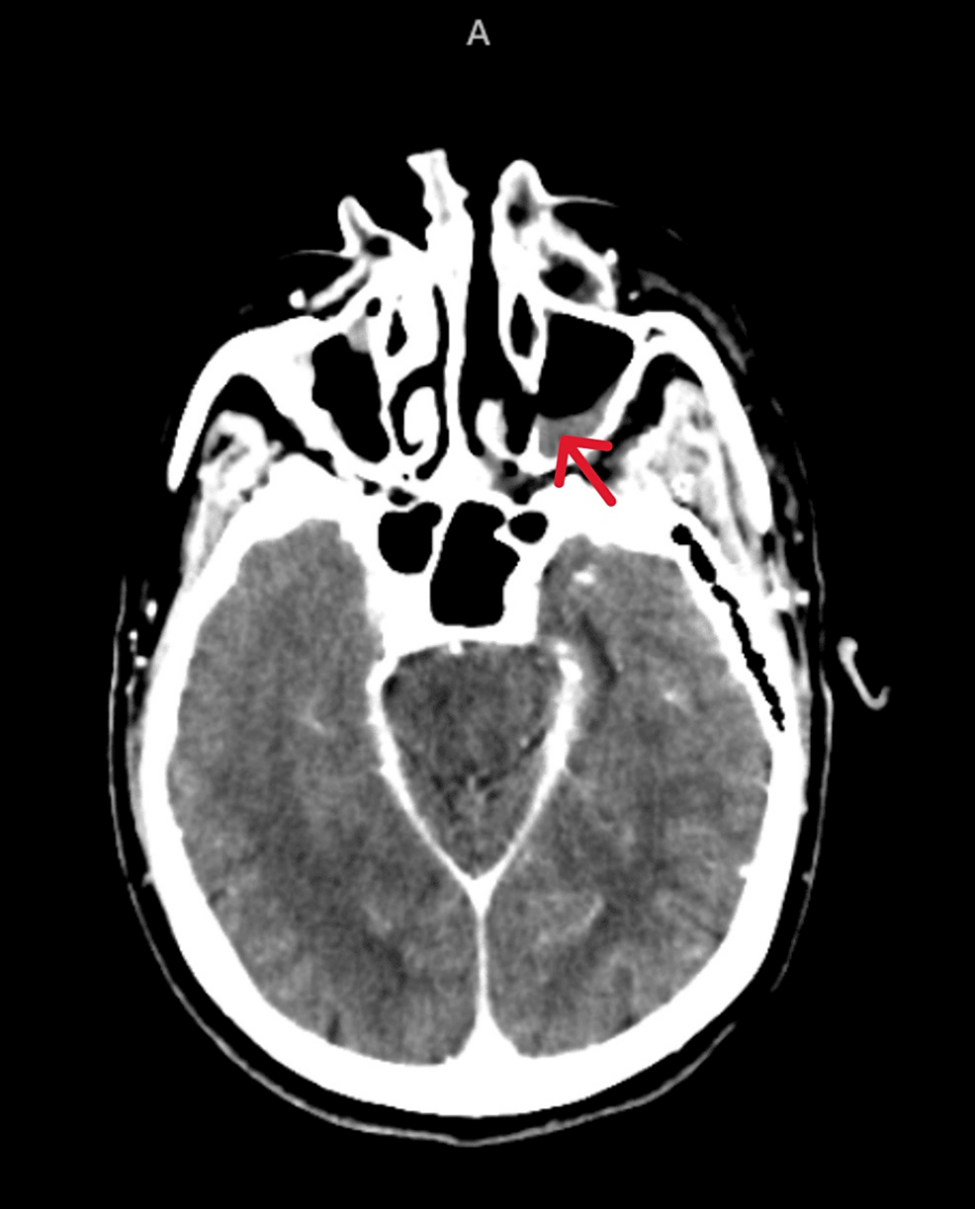
Contrast-enhanced computed tomography (CECT) brain and the orbits showing bilateral maxillary sinus opacification (Red arrow)

Her random blood sugar was extremely high. Complete blood count showed leukocytosis with neutrophil predominance. Her inflammatory markers were elevated with normal renal and liver function tests. Blood cultures taken at different intervals were sterile. Pus from the maxillary sinus showed mixed growth of *Pseudomonas* and coagulase-negative *Staphylococcus* species (Table 1). She did not have any evidence of diabetic hyperglycemic emergencies at presentation. Non-contrast computed tomography brain bone window (non-contrast CT (NCCT) brain) showed pansinusitis (Figures 1-2). A contrast-enhanced computed tomography brain (CECT brain) demonstrated bilateral maxillary sinus, left anterior and posterior ethmoidal, and frontal sinus opacification. Furthermore, there was no orbital or intracranial extension including thrombosis of the cavernous sinuses (Figure 3). There had been several practical issues in obtaining an MRI brain before the invasive interventions were carried on as that facility was not available in the hospital where the patient was being treated.

**Table 1:** Laboratory Parameters HbA1C: hemoglobin A1C; CRP: C-reactive protein; ESR: erythrocyte sedimentation rate; AST: aspartate transferase; ALT: alanine transaminase

Investigation type	Investigation results	Reference value
Random blood sugar	377 mg/dL	<200 mg/dL
Fasting blood sugar	172 mg/dL	70-100 mg/dL
HbA1C	8%	<5.6%
White blood cells	20.39 × 10^9^/L	4-11 × 10^9^ /L
Neutrophils	76.1%	40%-60%
Lymphocytes	15.5%	20%-40%
Hemoglobin	13.7 g/dL	13-17 g/dL
Platelets	357 × 10^9^/L	150-400 × 10^9^/L
CRP	210 mg/L	<6 mg/dL
ESR	92 mm/1^st^ h	<30 mm/1^st^ h
Serum creatinine	1 mg/dL	0.7-1.3 mg/dL
Serum sodium	140 mmol/L	135-145 mmol/L
Serum potassium	4.6 mmol/L	3.5-5.5 mmol/L
Serum ionized calcium	1.36 mmol/L	1.1-1.4 mmol/L
Serum magnesium	0.83 mmol/L	0.73-1.06 mmol/L
Serum chloride	102 mmol/L	101-109 mmol/L
Serum phosphorus	1.64 mg/dl	3.2-5.5 mg/dL
Serum iron	110 µg/dL	60-170 µg/dL
Total iron-binding capacity	382 µg/dL	240-450 µg/dL
AST	30 U/L	8-33 U/L
ALT	24 U/L	4-36 U/L
Albumin	26.6 g/L	35-52 g/L
Total bilirubin	7.42 µmol/L	5-21 µmol/L

**Figure 4:**
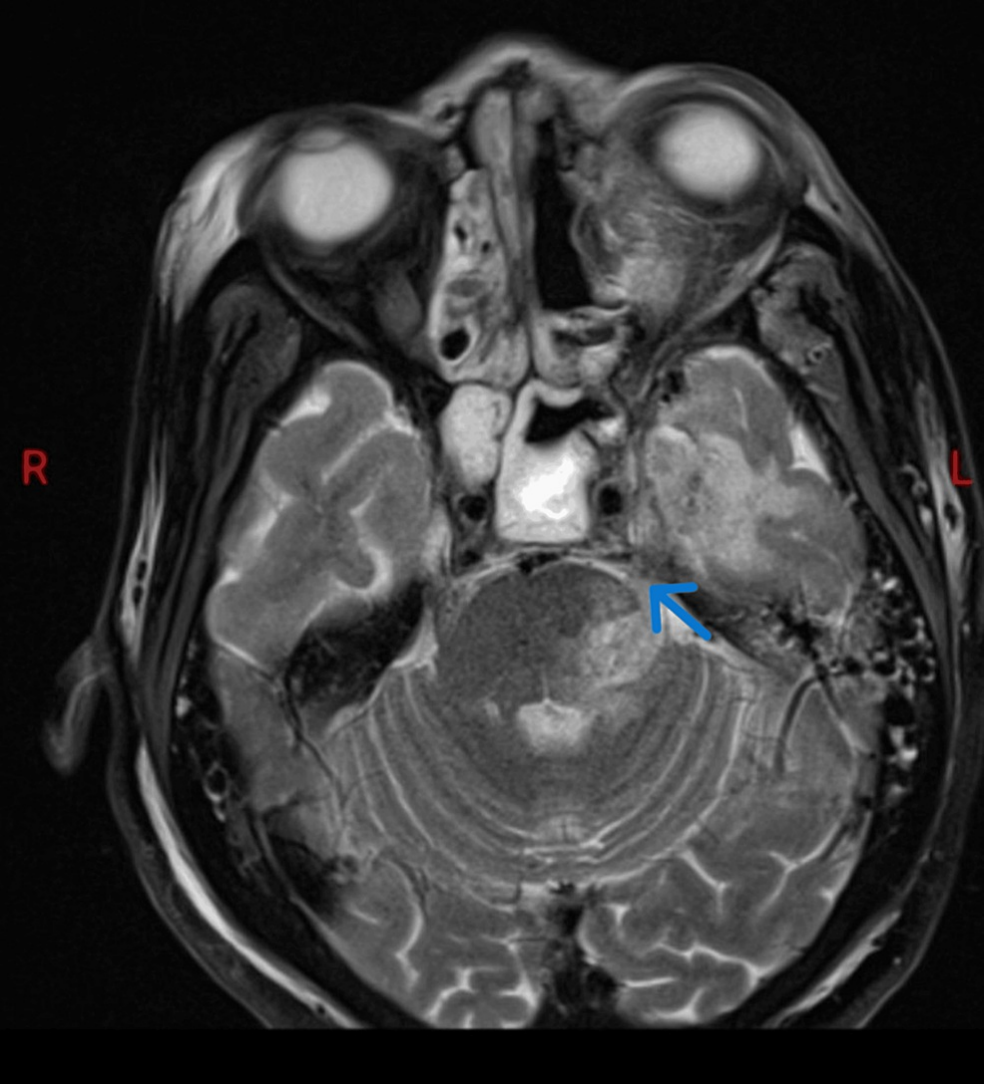
MRI brain T2 axial image shows thickening of the cisternal and cavernous portions of left trigeminal nerve (blue arrow) associated with T2 hyperintensity in the left hemipons and left middle cerebellar peduncle.

The postsurgical MRI brain showed thickening of the left trigeminal nerve with abnormal high signal intensity along the pathway of the trigeminal nerve which could be due to intracranial tracking with pansinusitis (Figure 4).

The patient underwent a left full-house functional endoscopic sinus surgery with right middle meatal antrostomy with left orbital decompression. She was found to have a necrosed left middle turbinate and unhealthy mucosa over the left side inferior turbinate, maxillary sinus, superior turbinate, anterior and posterior ethmoids, frontal recess, and right-side maxillary sinus with fungal debris and unhealthy mucosa in the posterior wall. Direct microscopy showed fungal filaments suggestive of zygomycetes. The fungal culture yielded *Rhizopus* sp. while histopathological analysis demonstrated fragments of necrotic tissue interspersed with spore and stubby broad hyphae.

She was commenced on IV amphotericin B (lipid formulation) at 3 mg/kg/day empirically which was continued for 85 days with gradual tapering of the dose. IV meropenem was continued for 18 days depending on the cultures and clinical response. Conservative treatment was done for the total ophthalmoplegia with left complete ptosis as the visual acuity of the left eye was not improving beyond 6/60 acuity with only a slight improvement of the ptosis. There was no significant improvement of the cranial palsies resulting in residual neurological deficits.

## Discussion

Rhinocerebral mucormycosis affects those with poor diabetes control. Altered glycemic status is favorable for fungus to act with angio-invasivity resulting in thrombosis leading to extensive necrosis. The enzyme ketoreductase in *Rhizopus *sp. provides the advantage of utilizing ketone bodies in diabetic ketoacidosis. It is also described that the high glucose environment along with reduced chemotaxis and phagocytosis impeding neutrophilic activity facilitates the proliferation of these otherwise harmless fungi [4,5].

The fungus spreads via blood vessels from the nasal cavity to the turbinate and palate and thereafter to the sinuses and retro-orbital space. Initially, the nasal inflammation may manifest as nasal obstruction, purulent rhinorrhea, facial pain, and epistaxis. This is followed by necrosis, a spectrum of chemosis, ptosis, proptosis, complex ophthalmoplegia, and complete blindness as a result of impairment of cranial nerves II, III, IV, and VI [6]. Single cranial nerve involvement is widely seen in diabetic patients. However multiple cranial palsies occurring together as extensive disease as in our patient is rare [7,8].

Reports of VII nerve palsy are sparse [9]. The reasons for facial nerve involvement are speculated to be the extension from the pterygopalatine fossa (a reservoir of Mucorales) to the inferior orbital fissure orbital apex and infratemporal fossa to the stylomastoid foramen from which the nerve exits. The proximity of the pterygopalatine fossa with myriad neurovascular structures has led to theories of perineural invasion. There is also vascular compromise due to edema that can also lead to ischemia of the facial nerve [9]. The facial nerve could be compromised by the spread of the disease through the eustachian tube or vascular connections of the middle ear [7,10].

Fungi are detected in tissues by histopathology with or without fungal culture. Although fungal culture aids diagnosis, it may often yield no growth unless the best yield is obtained at the necrotic and non-necrotic tissue interface. Thus, histological diagnosis is essential for diagnosis [11]. This patient had a positive yield of fungal growth. The PCR-based techniques performed on histologic specimens showing fungal elements can also confirm a mycological diagnosis. Computed tomography paves the way for the assessment of the extent of the disease. As in our patient sinus opacification is demonstrated by radiography. In advanced disease, there can be bony destruction with abscess formation and orbital involvement [5]. Magnetic resonance imaging as done in our patient provides clarification of the extent of the disease for surgical resection.

Mucormycosis requires an extensive combination of surgical debridement with parenteral amphotericin and rapid reversal of underlying predisposing factors. Antifungals alone are not adequate to contain the infection as blood vessels may be compromised by extensive thrombosis resulting in poor drug delivery [12]. Meticulous surgical debridement with removal of necrotic tissues and debulking infection is associated with improved survival. This may result in disfiguration and involvement of speech and swallowing. Various rehabilitative strategies are offered surgically and with prostheses [5].

Amphotericin B is the drug of choice with liposomal preparation preferred to the other preparation to deliver a high dose with less nephrotoxicity [13]. The commencement dose is 5 mg/kg/daily stepping up to 10 mg/kg/daily in severe disease. The duration of therapy is decided by the clinical and radiologic resolution of the active disease. Other possible therapies include combination antifungal therapy (amphotericin and echinocandins), deferasirox, an iron chelator, and hyperbaric oxygen of which the utility needs further research [13].

Early diagnosis and prompt treatment including correction of immunocompromised states and glycemic control are associated with good prognosis. The era, before effective antifungals and surgical options were available, was associated with higher mortality. The acute, rapidly destructive disease has more adverse outcomes and poor survival rates compared to long-standing diseases of insidious beginnings.

## Conclusions

This case report of poorly controlled diabetes mellitus highlights the importance of recognizing extensive multiple cranial nerve involvement (including a subtle seventh nerve palsy) in patients presenting with rhinocerebral mucormycosis. Histological and mycological diagnoses confirm the presence of the disease whilst imaging of the head with an MRI brain and sinuses will help determine the extension of the disease. Early detection and multimodal treatment including chemotherapy and debridement surgery are the preferred treatment options in such cases.
